# The Effects of Six Weeks of Endurance Training and CGRP Inhibition on Nrf2 and AKT Expression in the Hippocampal Tissue of Male Wistar Rats

**DOI:** 10.1155/2022/1610293

**Published:** 2022-08-31

**Authors:** Maryam Zare, Shila Nayebifar, Soheil Aminizadeh, Majid Vahidian-Rezazadeh

**Affiliations:** ^1^Department of Sport Sciences, Faculty of Educational Sciences and Psychology, University of Sistan and Baluchestan, Zahedan, Iran; ^2^Department of Physiology and Pharmacology, Afzalipour School of Medicine, and Physiology Research Center, Institute of Neuropharmacology, Kerman University of Medical Sciences, Kerman 78516-44438, Iran

## Abstract

**Purpose:**

To study the effects of a six-week endurance training protocol and calcitonin gene-related peptide (CGRP) inhibition on the nuclear factor erythroid 2-related factor 2 (Nrf2) and protein kinase B (PKB) or AKT expression in the hippocampal tissue of male Wistar rats. *Main Methods*. Building on a controlled experimental design with a posttest, 28 healthy Wistar male rats were randomly assigned to four groups (*n* = 7 per group), including *control*, *control+CGRP inhibition*, *endurance training*, and *endurance training+CGRP inhibition* groups. The training groups were trained for six weeks. Rats in the CGRP inhibition group received CGRP receptor antagonist daily (0.25 mg/kg) via intravenous (IV) injection. The Nrf2 and AKT (PKB) expression was measured using the real-time PCR technique.

**Results:**

In the endurance training group, Nrf2 expression in the hippocampal tissue was increased significantly more than in other groups (*P* < 0.05). There was also a significant increase in the AKT expression in the endurance training group compared to the control group (*P* = 0.048) and in the endurance training+CGRP inhibition compared to the control group (*P* = 0.012). In addition, there was no significant relationship between AKT (PKB) and Nrf2 (*r* = −0.27, *n* = 28, *P* = 0.16).

**Conclusion:**

Endurance training alone has been able to increase Nrf2 and AKT (PKB) mRNA levels in the hippocampal tissue, considering that endurance training had no significant effect on AKT and Nrf2 expression after adding to CGRP inhibition.

## 1. Introduction

Because of the increased oxygen demand and the presence of peroxidation-sensitive fat cells in the brain, it stands as one of the critical organs prone to damage by reactive oxygen species (ROS) [1]. This detrimental process can gradually lead to the inefficiency of the brain's antioxidant defense system [2]. ROS has been implicated in the pathogenesis of neurodegenerative diseases [3] such as amyotrophic lateral sclerosis (ALS), Parkinson's disease (PD), Huntington's disease (HD), and Alzheimer's disease (AD) [4].

Deoxyribonucleic acid (DNA) and proteins, among other living organisms, may be harmed under oxidative stress, leading to disrupted cellular processes. Under these conditions, specific cellular reactions must be activated to counteract the increased ROS levels and protect against oxidative damage [5]. Siciliano et al. were the first to report a link between exercise and oxidative stress more than 40 years ago [6]. While exercise affects the entire brain, the hippocampus is involved most. The hippocampus is a critical brain structure for memory and learning. Exercise has been shown to affect neurogenesis in the dentate gyrus of the hippocampus, which in turn increases synapse flexibility and the number of neurons. Thus, exercise improves the antioxidant capacity of the brain, especially in the hippocampus [7]. It increases ROS levels through various mechanisms that activate nuclear factor erythroid 2-related factor 2 (Nrf2). Previous literature indicated exercise strategies like aerobic training [8], resistance exercise [9], and herbal medicine [10] which can successfully target neuroplasticity in the hippocampus.

Increased ROS levels can be postulated to contribute significantly to Nrf2 activation during exercise [11]. Typically located in the cytosol [12], Nrf2 encoded by nuclear factor erythroid 2-like 2 (NFE2L2) in humans is a major regulator of antioxidant defense. This transcription factor regulates the expression of more than 200 cell-protecting genes [13]. Under normal physiological conditions, Nrf2 is inhibited by Kelch-like ECH-associated protein 1 (KEAP1). Nrf2 also interacts with the cullin-3 E3-ubiquitin ligase (Cul3) [14], which facilitates the Nrf2 degradation. When exposed to oxidative stress, Nrf2 is cleaved from Keap1, transported into the nucleus, and binds to the antioxidant response elements (ARE) region by forming heterodimers with small MAF proteins [15]. As such, it translates to detoxifying enzymes such as glutathione synthetase (GSS), glutathione reductase (GR), thioredoxin (TRX), thioredoxin reductase (TRR), and peroxiredoxin (PRX) to prevent oxidative stress.

In the brain, the protein kinase B (PKB) or AKT pathway mediates the effects of exercise [14]. Serine/threonine kinase AKT regulates various cellular activities, including proliferation, growth, survival, apoptosis, metabolism, transcription, and protein synthesis [16]. ROS has a direct role in activating the phosphoinositide 3-kinase (PI3K) enzyme [14]. AKT (PKB)/PI3K signaling pathway regulation significantly reduces ROS production and cell apoptosis status [17]. AKT (PKB)/PI3K pathway inhibition prevents exercise-induced synaptic flexibility and neurogenesis in the dentate gyrus of the hippocampus. It has been shown that six weeks of high-intensity interval training (HIIT) contribute to neurogenesis and increase antioxidant defense by reducing oxidative stress in the hippocampus [7]. PI3K prompts the recruitment of downstream molecules, including AKT (PKB), by phosphorylating phosphatidylinositol 4,5-bisphosphate (PIP2) and producing phosphatidylinositol (3,4,5)-trisphosphate (PIP3). AKT (PKB) activation leads to sequential activation and transcription of target genes, including glycogen synthase kinase3 (GSK3) [14]. PI3K induces cell viability by activating AKT (PKB) phosphorylation and Nrf2 nucleus transfer [18]. The Nrf2 transcription factor is regulated directly by the glycogen synthase kinase 3*β* (GSK3*β*) in the AKT (PKB)/PI3K pathway. GSK3*β* regulates nuclear output and Nrf2 degradation. However, phosphoglycogen synthase kinase 3*β* (*P*-GSK3*β*) prevents this through Nrf2 phosphorylation and, thus, inhibits Nrf2 degradation. Eventually, Nrf2 is transferred into the nucleus and exerts the antioxidant effects of oxidative stress by enhancing the transcriptional expression of downstream genes [19].

Exercise activates Nrf2 via the AKT/PI3K pathway and facilitates its transfer to the nucleus to regulate the antioxidant defense system [20]. AKT (PKB)/PI3K and Nrf2/Keap1 pathways are significantly regulated to decrease ROS levels by elevating antioxidant levels, hence causing alterations in metabolism or other mechanisms [21]. Calcitonin gene-related peptide (CGRP) is a 37-amino acid neuropeptide, which is produced by the intermittent binding of the calcitonin (CT) on chromosome 11 [22]. CGRP can activate the neuroprotective process in the brain by reducing oxidative stress [23]. However, it is not clear whether the inhibition of CGRP secretion, which occurs during specific diseases or physical activity, can affect the level of oxidative stress in the hippocampus and possibly lead to neurogenesis. This, indeed, requires further research. Accordingly, the present study is aimed at investigating the effects of six weeks of endurance training and CGRP inhibition on the expression of Nrf2 and AKT (PKB) genes in the brain tissue of male Wistar rats.

## 2. Materials and Methods

### 2.1. Experimental Animals

The study adopted a controlled experimental design with a posttest and was performed in Kerman Physiology Research Center. The Ethics Committee of the University of Sistan and Baluchestan approved the study protocol. At first, thirty-two rats were included in the study, 8 in each group. However, during the program, one died and dropped from each group. Finally, twenty-eight rats were remained weighing 200.14 ± 7.91 g (aged eight weeks), which were prepared from Kerman Physiology Research Center. They were kept under controlled conditions (light/dark cycle: 12/12 hours, temperature: 22 ± 2°C) and had free access to water and healthy and standard food (pellets). After two weeks of adaptation, the animals were randomly assigned to four groups (*n* = 7 per group), including a control group, a control group with CGRP inhibition, an endurance training group, and an endurance training group plus CGRP inhibition.

### 2.2. CGRP Inhibition

CGRP inhibition in the relevant group was performed through intravenous injection of CGRP receptor antagonist (compound) (BIBN4096BS from Merck Company, Darmstadt, Germany) using a 1 ml insulin syringe at a dose of 0.25 mg/kg every day and few hours before the treadmill exercise [24]. BIBN4096BS is a potent receptor antagonist that can inhibit CGRP activation and synthesis in the brain [25].

### 2.3. Exercise Protocol

The endurance training prescribed in this study included running on a treadmill with a zero slope for six weeks, five days a week. During the first two weeks, the training groups ran on the treadmill at a speed of 15 meters per minute for 20 minutes. The intensity (speed) and volume (duration) of training were designed based on the gradual increase of workload [26], whose details are provided in Table 1. Control groups were kept in cages without training during the training period. However, to create similar conditions in terms of treadmill stress, all rats were placed on a silent treadmill and shocked electrically in each session.

### 2.4. Sampling

The rats were anesthetized using a combination of xylazine (10 mg/kg) and ketamine (90 mg/kg) 48 hours after the last training session. After the brain was incised with a surgery razor, the hippocampal tissue was extracted in a completely sterile environment, immediately frozen in liquid nitrogen, and stored at −80°C for real-time PCR measurements.

### 2.5. Real-Time PCR

A Hielscher sonicator (Hielscher H200, Germany) was used to dissect and homogenize about 50 mg of the hippocampal tissue at a specific lysis buffer. After the total RNA was extracted based on the kit protocol (Bio Basic Kits, 50 preps, Canada), complementary DNA (cDNA) was synthesized from the extracted RNA (500 ng). A cDNA synthesis kit (Pars Tous Aminsan Company, Iran) was used per the manufacturer's instructions for synthesis. We used specific primers in real-time PCR measurements, as reported in Table 2. ABI StepOnePlus™ Real-Time PCR System was used to perform real-time PCR reactions, which contained 10 *μ*l SYBR green, cDNA (100 ng), and forward and reverse primers (1 *μ*l of each primer). The reaction volume was reached to 20 *μ*l by distilled Dnase-free water, and the annealing temperature was determined according to each primer Tm and gradient PCR.

The thermal protocol in real-time PCR is as follows: 95°C (10 min), 95°C (15 sec), and annealing temperature (30 sec) at 40 cycles. After the thermal cycles were completed, the melt curve analysis was performed. We used 18S as a reference gene to measure relative gene expression and control the product's specialized replication by melting curve analysis. The relative expression of genes was determined using the 2^−∆∆Ct^ method.

### 2.6. Statistical Analysis

The normality of data distribution was confirmed using the Shapiro-Wilk test. The homogeneity of variances was checked and confirmed using Levene's test. Besides, the one-way ANOVA test was employed for intergroup comparison. Lastly, the Tukey test determined the differences between groups. Data were analyzed in SPSS software, ver. 24, and *P* values smaller than 0.05 were considered significant in all tests.

## 3. Results

**Figure 1:**
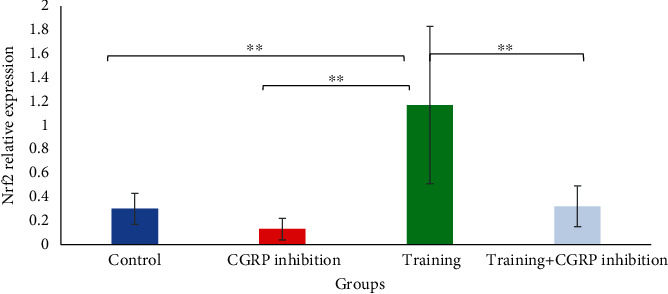
Nrf2 gene expression comparison in hippocampus between groups. ^∗∗^Significant difference compared to training group (*P* < 0.05).

**Figure 2:**
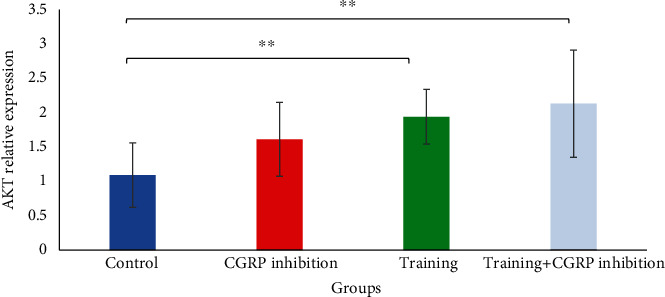
AKT gene expression comparison in hippocampus between groups. ^∗∗^Significant difference compared to control group (*P* < 0.05).

**Figure 3:**
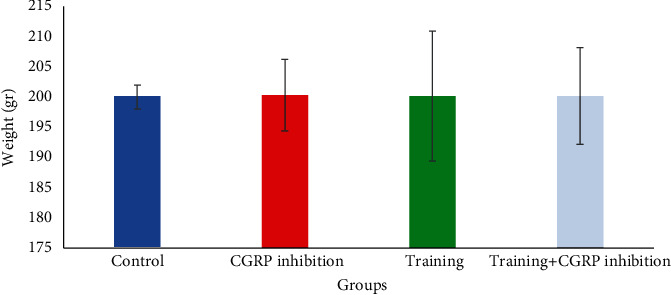
Weights (mean ± SD) of rats in groups.

Figure 1 shows that there was a significant increase in Nrf2 expression level in the endurance training group compared to other groups (*P* < 0.05). Besides Figure 2, there was a significant increase in AKT expression in the endurance training group compared to the control group (*P* = 0.048) as well as in the endurance training group+CGRP inhibition group compared to the control group (*P* = 0.012) (Table 3). Descriptive data concerning the weight of the rats are reported in Table 4 and Figure 3.

### 3.1. AKT and Nrf2 Correlation

The Pearson correlation coefficient was used to investigate the correlation between AKT (PKB) and Nrf2 indices. At a significance set at *P* < 0.05, no significant relationship was found between the two indices (*r* = −0.27; *P* = 0.16; *n* = 28).

## 4. Discussion

The present study is aimed at determining the effect of six weeks of endurance training and CGRP inhibition on Nrf2 and AKT (PKB) expression in the hippocampal tissue of male Wistar rats. Findings revealed no significant change in hippocampal AKT (PKB) expression in the endurance training+CGRP inhibition group compared to the CGRP inhibition group. Several factors can be involved here. First, certain brain parts, such as the cerebral cortex, cerebellum, and brainstem, are more responsive to exercise. In other words, the activation of brain regions during exercise may not be the only factor influencing CGRP expression. Some studies, for example, indicate that while the cerebellum is more active, it does not show an increase in CGRP expression during exercise [27]. Thus, there is no linear match between the CGRP expression in the hippocampus and the whole brain [28]. Accordingly, activation during training cannot be the only influential factor [27]. Second, the nonintegration of CGRP expression in the brain is attributed to the different responses of different brain parts to exercise [29]. Third, some exercise-induced secretory factors, including lactate, may increase CGRP expression during exercise in the brain [30]. Exercise intensity is also an important factor that increases CGRP [31], while the intensity in present protocol was moderate. However, the training alone could increase AKT expression compared to control.

Consistent with the present study, Jung and Kim investigated the effect of a 4-week treadmill training protocol that activated the PI3K/AKT (PKB) pathway on improving motor function and memory in paralyzed rats. By activating the PI3K/AKT (PKB) pathway, the treadmill training increased hippocampal cell proliferation and neurogenesis (through increased synaptic properties), thereby improving motor function and memory in rats with cerebral palsy [32]. Kang and Cho investigated the effect of 12 weeks of treadmill training on PI3K/AKT (PKB)/mTOR, autophagy, and hyperphosphorylation of the tau protein in the cortex of the Alzheimer's mice, showing that exercise increased cerebral AKT (PKB) expression. They discussed that higher AKT (PKB) activity leads to improved survival, neuroprotective effect, and inhibition of GSK-3*β* activity [33]. In another study, Fang et al. investigated the impact of five consecutive days of treadmill training (15 m/min for 30 minutes per day) on the PI3K/AKT (PKB) signaling pathway in the rat hippocampal tissue. They found that treadmill training modulates oxidative stress in rat hippocampal tissue by significantly enhancing the PI3K/AKT (PKB) signaling pathway [34].

As the most significant downstream PI3K factor, AKT (PKB) can regulate many PI3K downstream factors such as kinases and transcription factors for cell function. For example, the nuclear transfer of Nrf2 occurs to control the antioxidant defense system via the PI3K/AKT (PKB) pathway [35]. There is ample evidence that exercise-induced physiological adaptations prompt the expression of different genes [36]. The lack of relationship between these genes may be attributed to the fact that AKT (PKB) activated other downstream factors when provoked by endurance training. On the other hand, AKT (PKB) overexpression activates Nrf2 [37]. It is also possible that the exercise protocol did not respond to AKT (PKB) expression in a way that could lead to Nrf2 activation in the oxidative stress pathway.

The present study examined the role of the CGRP peptide as it is both widely distributed in the brain and is involved in processes that cause changes in hippocampal excitability, such as learning, memory, and oxidative stress [38]. CGRP can play a role in ROS suppression [39]. There is currently little information about the impact of physical activity on cerebral CGRP levels and its positive effects on cognition [40]. Although the brain is shown to have higher levels of CGRP release during exercise, there is still no documented evidence of CGRP's physiological role during exercise [41, 42]. Nonetheless, some studies have shown that physical activity, including aerobic exercise can increase CGRP [31, 40]. It has been demonstrated that CGRP can benefit cognitive function by improving neurogenesis and synaptic hippocampus transmission [28]. CGRP may, to some extent, play a neuroprotective role through the AKT (PKB)/mTOR signaling pathway [43]. It can be inferred that even AKT (PKB) is upregulated with CGRP inhibition. This upregulation is shown here insignificantly.

The present study also showed no positive effect of adding training to CGRP inhibition on Nrf2 expression. It seems may be the short duration of training (6 weeks) or exercise type (continuous nature) and the intensity (moderate load) were not enough to stimulate the gene expression. However, a significant increase in the Nrf2 hippocampal expression in the endurance training group alone compared to other groups was achieved. In this regard, Aboudeya et al. investigated the impact of exercise on Nrf2 expression and oxidative stress in the hippocampal tissue of rats with Alzheimer's disease. Their findings were consistent with the results of the present study, suggesting that physical activity increased Nrf2 expression in the hippocampal tissue. In fact, exercise could exert neuroprotective effects in Alzheimer's disease by regulating Nrf2, leading to the improved antioxidant capacity of the hippocampal tissue [44]. Similarly, Tutakhail et al. investigated the impact of a 3 to 7-week treadmill training protocol at three intensities on Nrf2 and HO-1 expression in the hippocampus, cortex, hypothalamus, and pain threshold in adult rats. They found that only high-intensity prolonged aerobic exercise increased Nrf2 protein levels in the hippocampus [45]. Similarly, four weeks of treadmill training are shown to overregulate and have protective effects on Nrf2 [46].

Soleimani et al. investigated the effect of a one-month progressive treadmill training program conducted for 10-30 minutes at a speed of 1-25 m/min on the brains of rat models with Alzheimer's disease. They studied the Nrf2 expression in rat hippocampal tissue and concluded that treadmill training upregulated the Nrf2 expression. However, this increase was not significant [47], which is inconsistent with our results. In previous research (Doudly, Krisuy), exercise intensity and the stress related to the treadmill vehicle have been shown to affect the biomarker contents. However, the intensity applied in the present protocol was moderate, and to normalize the stress, all rats were put on the treadmill [48, 49].

Despite the lack of research with human models, basic animal models strongly support the idea that exercise is a significant protective factor against neuronal damage for several reasons. Exercise reduces oxidative stress and counteracts brain damage. Indeed, the brain's response to exercise regulates the antioxidant system and modulates oxidative stress. In contrast, little exercise and mobility are associated with increased oxidative stress, which can cause neurodegeneration, especially in the hippocampus [50, 51]. Findings from various studies reveal that endurance training increases antioxidant activity [52] and positively affects hippocampal neurogenesis [53]. This being said, it can be concluded that endurance training in the training groups without CGRP inhibition may contribute to hippocampal neurogenesis by modulating the antioxidant defense factors, i.e., Nrf2 and AKT(PKB), through oxidative stress modulation (although oxidative stress levels were not measured in the present study). On the other hand, there was no significant correlation between Nrf2 and AKT (PKB) genes in the present study.

Overall, it can be stated that endurance training could not improve antioxidant defense by upregulating Nrf2 and AKT (PKB) levels in the hippocampal tissue after CGRP inhibition. So, to consider exercise as a complementary therapy in neurodegenerative diseases, further research is warranted to increase knowledge concerning the pathways involved in the oxidative stress process by manipulating the duration and intensity. Moreover, researchers need to investigate the effect of exercise on protein expression involved in the oxidative stress process and ROS levels.

## 5. Research Limitations

One of the limitations of the present study is the researcher's inability (financial constraint) to measure ROS levels and those of other proteins and downstream AKT molecules in the ROS-dependent messaging pathway. Information about these levels could help understand more accurately the mechanisms underlying endurance training. Notably, the authors were unable to utilize protein detection procedures such as Western blotting or immunohistochemistry to measure Nrf2 and AKT in the hippocampus.

Lastly, the control rats were not treated with the vehicle to control for stress among samples. Moreover, previous research reports that the role of treatment on gene transcription may not fully predict protein synthesis and protein circulating levels. However, mRNA levels indicate a significantly positive correlation with protein expression (40%) [54, 55].

## Figures and Tables

**Table 1:** Endurance training protocol on a treadmill with zero slope.

Weeks	1 and 2	3	4	5	6
Speed (m/min)	15	22	25	27	27
Time duration (min)	20	35	40	45	50

**Table 2:** The primers' sequences used to perform real-time PCR.

Gene name	ac. no.	Forward primer (5′ to 3′)	Reverse primer (5′ to 3′)	Product size (bp)
18S	XR006521717.1	GCAATTATTCCCCATGAACG	GGCCTCACTAAACCATCCAA	122
Nrf2	XM032903520.1	CACATCCAGACAGACACCAGT	CTACAAATGGGAATGTCTCTGC	118
AKT	XM032908539.1	CTGGGTTACCCCGGTGTGT	GCACATCCGAGAAACAAAA	124

**Table 3:** Comparison of gene expression changes in groups.

Variable (∗)	Training + CGRP inhibition (mean ± SD)	Training (mean ± SD)	CGRP inhibition (mean ± SD)	Control (mean ± SD)	*F*	*P* ANOVA	Multiple comparisons (Tukey)		
							Groups	Mean differences	*P* value
Nrf2 (relative expression)	0.32 ± 0.17	1.17 ± 0.66	0.13 ± 0.09	0.30 ± 0.13	11.93	^∗^<0.001	Control vs. train	0.86	^∗^<0.001
							Train vs. CGRP	1.03	^∗^<0.001
							Train vs. train +CGRP	0.84	^∗^<0.001
AKT (relative expression)	2.13 ± 0.78	1.94 ± 0.40	1.61 ± 0.54	1.09 ± 0.47	4.40	^∗^0.01	Control vs. train	0.84	^∗^0.04
							Control vs. train+CGRP	1.03	^∗^0.01

**Table 4:** Means and standard deviations of rats' weights in study groups.

Variable	Training+CGRP inhibition(mean ± SD)	Training (mean ± SD)	Control (mean ± SD)	CGRP inhibition (mean ± SD)
Weight (gr)	200.14 ± 8.01	200.14 ± 10.77	200.00 ± 2.00	200.29 ± 5.93

## Data Availability

All data may be made available from the corresponding author upon reasonable request.
